# Practical surgical technique using the SMISS approach for lip reduction in involuted lip infantile hemangiomas

**DOI:** 10.1186/s12887-024-04838-4

**Published:** 2024-05-28

**Authors:** Qianyi Chen, Shih-Jen Chang, Wei Gao, Lei Chang, Yajing Qiu, Xiaoxi Lin

**Affiliations:** Department of Plastic and Reconstructive Surgery, Shanghai Ninth People’s Hospital, School of Medicine, Shanghai Jiao Tong University, Zhizaoju Road, Shanghai, 200011 China

**Keywords:** Infantile hemangioma, Lip hemangioma, Lip reduction, Facial surgical procedure, Propranolol

## Abstract

**Background:**

Lip infantile hemangiomas tend to show less volumetric regression and are more susceptible to visible sequelae in the involuted stage. Some of them still require surgical management after propranolol therapy. This study aimed to evaluate the efficacy and safety of the Stepwise, Multi-Incisional, and Single-Stage (SMISS) approach applied to lip reduction for those with involuted lip hemangiomas.

**Methods:**

A retrospective review was performed to evaluate patients with lip hemangioma who received previous propranolol treatment and underwent the aforementioned procedure. Demographic characteristics, lesion morphology, and medical history were reviewed. The Visual Analog Scale was applied to assess the postoperative appearance. Complications within 12 months postoperatively were recorded.

**Results:**

A total of 18 patients with lip hemangioma were eligible. All patients received oral propranolol therapy before surgery, with treatment duration ranging from 6.0 to 23.0 months. Their age at surgery ranged from 2.5 to 9.0 years. The median Visual Analog Scale scores were 8.0, ranging from 4.0 to 10.0. No severe complications were reported.

**Conclusions:**

This modified technique based on the SMISS approach has proven reliable and effective in improving the aesthetic outcome for involuted lip infantile hemangiomas. Practical surgical techniques still play an important part in the propranolol era.

## Background

Infantile hemangioma (IH) is the most common benign tumor in infancy, and an incidence as high as 4.5% has been previously reported in a prospective study [1]. Even though some IHs resolve spontaneously, approximately 10-15% of cases develop complications such as ulceration, obstruction, or disfigurement, warranting immediate therapy during the proliferative stage [2], among which ulceration is the most frequent complication [3]. The ulcerations of IHs had a predilection for some specific locations, especially the lip [4]. Ulcerations in lips may have deleterious effects, including pain, secondary infections, difficulties in feeding, irritability, and scarring after healing, and these issues could aggravate parental stress [5].

In the involuting phase, the lesions located on the lips showed significantly less volumetric regression and were more susceptible to severe sequelae [6]. Since the lip is considered a cosmetically sensitive region, both the residual lesions and the scar formation in lips would yield conspicuous deformities, which tends to increase the risk of social inadequacies during the preteen years [7]. Taken together, even in the propranolol era, operative intervention is still necessary for a certain number of patients with lip IHs. However, the literature on the surgical management of lip IHs during the involutional phase is rare. Based on the Stepwise, Multi-Incisional, and Single-Stage (SMISS) approach [8], we hereby propose a novel paradigm of surgical treatment for lip IHs with complications.

## Patients and methods

### Study population

A retrospective review was performed to evaluate patients who underwent surgical lip reduction (by the SMISS approach) for involuted lip IH from January 2018 to December 2021. Surgical resection and reconstruction were performed by a single surgeon (X.L.). This study was approved by the ethics review board at the Shanghai Ninth People’s Hospital. Inclusion criteria were: i) patients diagnosed with deep or mixed lip IH according to the International Society for the Study of Vascular Anomalies (ISSVA) diagnostic criteria; ii) patients who underwent lip reduction surgery (by the SMISS approach) after 1 year of age; and iii) patients whose medical records contained sufficient information regarding demographic characteristic, lesion morphology, previous treatment before the surgery, and follow-up photographs (Follow-up visits were conducted at 6 and 12 months after the surgery.). Patients were excluded if adequate follow-up was not obtained.

### Study design and outcome measures

This was a retrospective study to evaluate the effect of SMISS approach on lip reduction surgery in involuted IH patients. Researchers first reviewed the archives of all eligible patients to collect demographic data, hemangioma features, and prior treatments. Then, follow-up photographs taken 12 months after the surgery were reviewed by 3 independent clinicians (Q.C., S.C., and Y.Q.), and the Visual Analog Scale (VAS) was used to evaluate cosmetic improvement. VAS was described in detail by McCormack et al. [9]. In brief, 0 indicates no cosmetic improvement, whereas 10 indicates the best imaginable outcome. Complications and sequelae after the surgery were also monitored.

Statistical analyses were performed using SPSS (version 17.0; IBM Corp., Armonk, NY, USA). The Mann-Whitney U test was used to analyze variation in VAS score between the upper and lower lip group. The significance threshold was set at *P* < 0.05.

### Operative technique

**Fig. 1:**
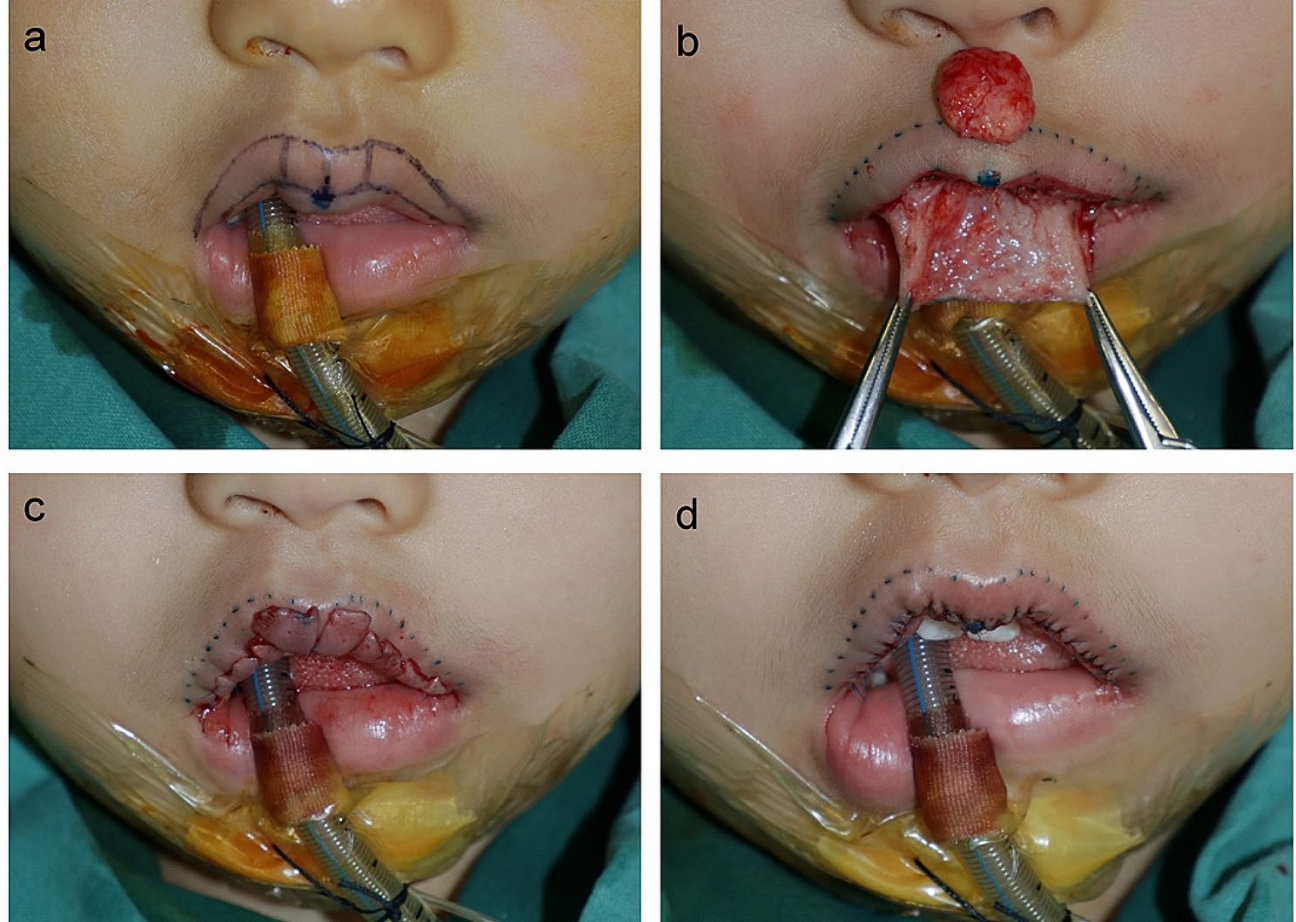
Surgical procedures of hemangioma excision and lip reduction on the upper lip. **a**, Defining the labial margin, the central point of the upper lip, and the wet-dry junction (the incision). **b**, After the tissue undermining was performed underneath the labial mucosa layer, the deep hemangioma was removed. **c**, The mucosal flap was split into multiple segments and the temporary suture was performed. **d**, After adjusting the length, orientation, and shape of each segment, the excision and the wound closure were stepwise performed

**Fig. 2:**
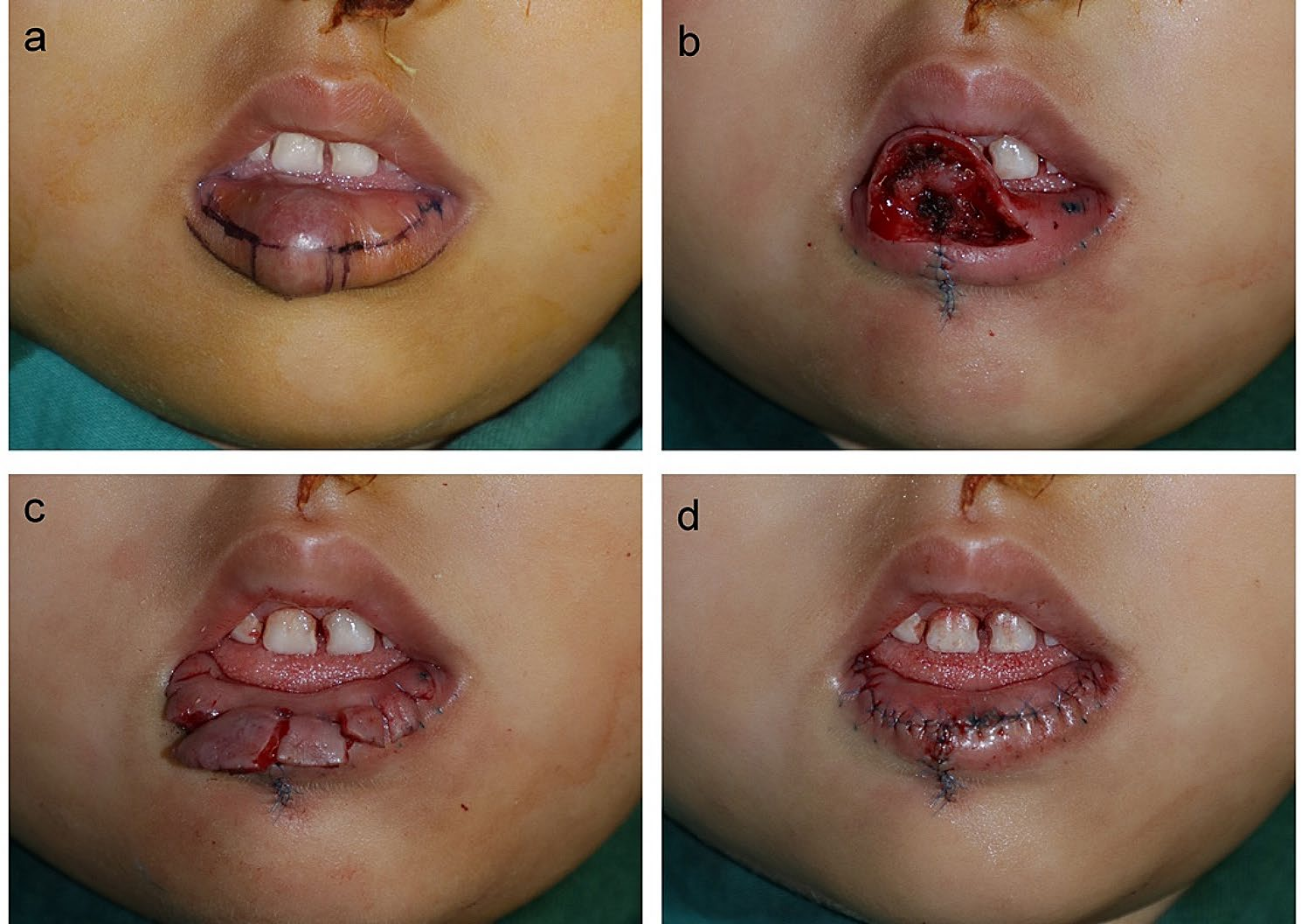
Surgical procedures of hemangioma excision and lip reduction on the lower lip. **a**, Defining the labial margin, the border of the lesion, and the wet-dry junction (the incision). **b**, The horizontal retraction of the lip was performed first. After that, the tissue undermining was performed underneath the labial mucosa layer, and the deep hemangioma was removed. **c**, The mucosal flap was split into multiple segments and the temporary suture was performed. **d**, After adjusting the length, orientation, and shape of each segment, the excision and the wound closure were stepwise performed

Under general anesthesia, the wet-dry junction was entirely and precisely marked using sterile methylene blue (Figs. 1A and 2A). The incision was made along this marked curve. Starting from the incision, tissue undermining was performed underneath the labial mucosa layer. The extent of undermining must cover the planned resected area, differing in each patient. Feeding and draining vessels were coagulated when encountered. For localized IHs, the subcutaneous lesions which were defined and did not affect the orbicularis oris muscle were completely excised along the boundary. After tumor removal, the adjacent tissue should be trimmed as appropriate (Figs. 1B and 2B). However, some deep lesions were diffuse, involving almost the whole upper or lower lip or disrupting the orbicularis oris muscle. Under this circumstance, a debulking procedure was needed instead of complete tumor removal. Thus, a wedge-shaped resection of the subcutaneous tissue was performed. The volume of surgical excision was tailored to each patient to avoid the risk of overreduction.

To improve the thickened appearance caused by IH expansion, the outer layer of the lip was partially resected as well. Generally, lip enlargement was characterized by vertical and/or horizontal lengthening.

In the context of vertical elongation, the SMISS approach was applied. The debulking procedure was performed for the labial mucosa area because the tissue expansion effect obviously occurred in the mucosa and there was no substitute for vermilion when the defects of it happened. Due to the aesthetic consideration, the volume of excision was determined depending on the thickness of the patient’s unaffected lip. Then, the surgeon marked the multiple vertical incisions on the mucosal flap at similar intervals. This flap was split into segments as designed and full-thickness suturing was performed to anchor the endpoint of each incision temporarily. In this manner, the entire flap can be stepwise divided into several segments (Figs. 1C and 2C). Once the temporary fixing of all cutting points was accomplished, one of the segments (usually beginning from the central to the lateral) was cut off and the wound was closed using 6 − 0 absorbable sutures. In this process, the surgeon could adjust the shape of the resection margin appropriately to make it match the natural contour of the normal lip. This step was repeated until the final incision presented a symmetrical appearance (Figs. 1D and 2D).

**Fig. 3:**
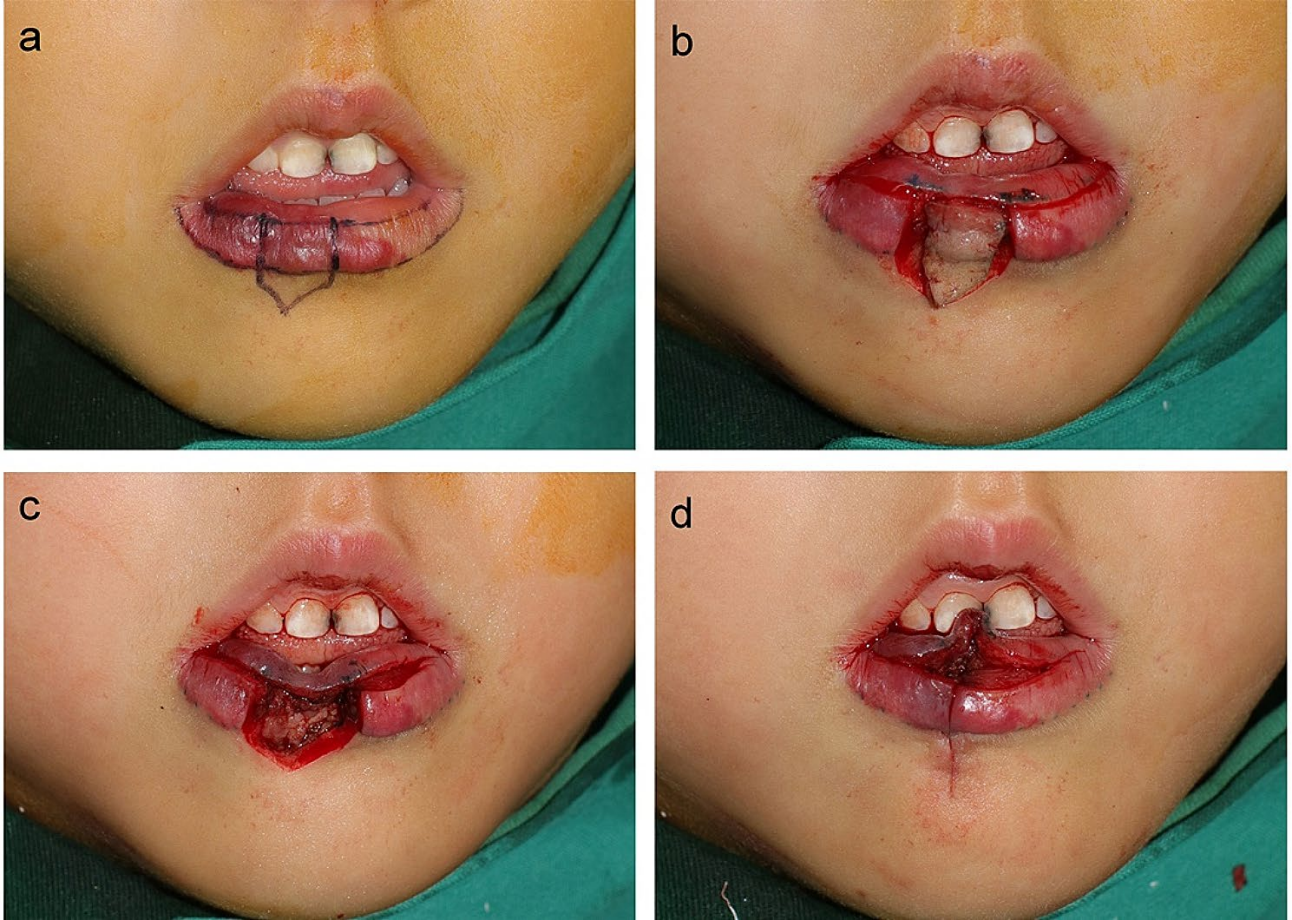
Surgical procedures of partial hemangioma excision and horizontal shortening of the lower lip. **a**, Defining the labial margin and the wet-dry junction (the incision). Preoperative markings were drawn for the pentagon-shaped resection. **b-d**, The incision on the wet-dry junction and a vertical, pentagon-shaped, and full-thickness resection was performed. The closure was performed layer by layer

If the horizontal lengthening existed simultaneously, it was dealt with before the vertical shortening. It was corrected by horizontal retraction of the lip through a vertical, pentagon-shaped, and full-thickness resection (Fig. 3A, B), which was designed on the superficial lesion of the vermilion, such as the erythema or the scar formed after ulceration healed. The incision was extended to the surrounding normal skin to minimize the dog-ear deformity and the closure was performed starting from the orbicularis oris layer, followed by the subcutaneous and cutaneous one (Fig. 3C, D). For IHs on the lower lip, special attention was paid when suturing the subcutaneous layer below the vermiliocutaneous junction to preserve or rebuild the mentolabial sulcus. This was achieved by local transverse shortening of the subcutaneous tissue in the aforesaid area, producing additional tension during the flap advancement.

Lastly, simple interrupted suturing using 7–0 nonabsorbable sutures was performed on surface layer of the incisions. The compression dressings were applied to the nonvermilion area of the affected lip for 24 h to restrain its movement.

## Results

A total of 18 cases, aged from 2.5 to 9.0 years (average, 4.4 ± 1.0 years) at the time of surgery, with involuted lip IHs were included in this study. The clinical characteristics were summarized in Table 1. Among them, four had lesions involving the upper lip and 14 on the lower lip. All patients received oral propranolol therapy before surgery, with treatment duration ranging from 6.0 to 23.0 months. 10 of them also underwent corticosteroid injection, laser, or sclerotherapy.

**Table 1:** Clinical characteristics of the patients

Patient Demographics	
Age at surgery, year	
Mean (SD)	4.4 (1.9)
Range	2.5-9.0
Gender, n (%)	
Male	3 (16.7%)
Female	15 (83.3%)
**Lesion Characteristics**	
Location, n (%)	
Upper lip	4 (22.2%)
Lower lip	14 (77.8%)
Lesion type, n (%)	
Superficial	0
Deep	0
Mixed	18 (100%)
Morphology, n (%)	
Diffused	6 (33.3%)
Localized	12 (66.7%)
History of ulceration, n (%)	6 (33.3%)
Propranolol treatment duration, months	
Mean (SD)	9.4 (4.2)
Range	6.0–23.0
**Postoperative Evaluation**	
VAS^a^	
Median (Range)	8.0 (4.0–10.0)
Cronbach α value	0.7
Sequelae, n (%)	
Scar hypertrophy	1 (5.6%)
Follow-up, months	
Mean (SD)	18.5 (6.6)
Range	12.0–30.0

a. VAS, Visual Analog Scale

**Fig. 4:**
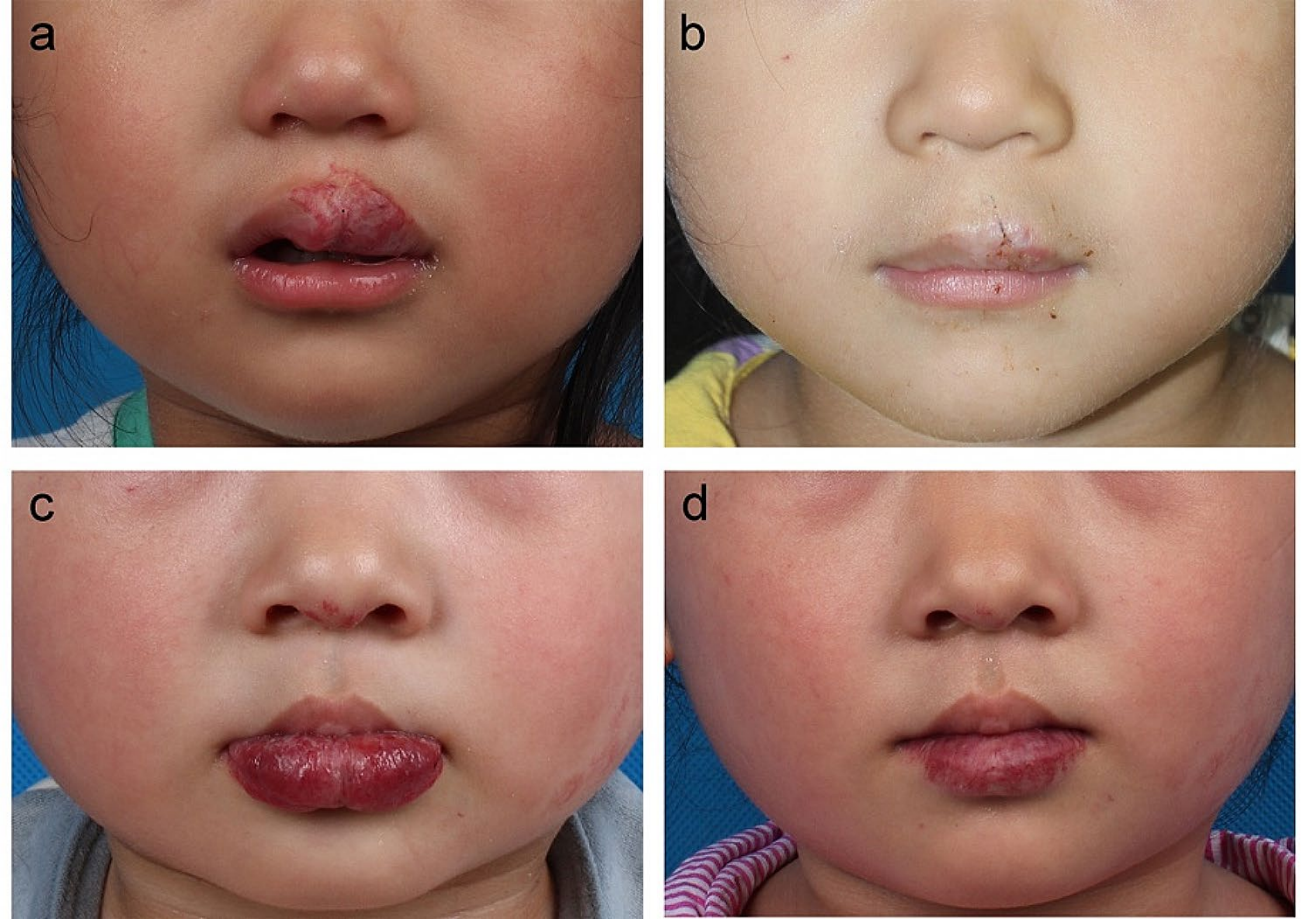
The patients underwent upper or lower lip reduction by the SMISS approach. **a-b**, A 3-year-old (at surgery) female patient with upper lip IH. **a**, Preoperative view. **b**, Twelve-month postoperative result. **c-d**, A 3-year-old (at surgery) female patient with lower lip IH. **c**, Preoperative view. **d**, Sixteen-month postoperative result

The follow-up duration was 12.0 to 30.0 months (average, 18.5 ± 6.6 months). No major complications, such as infection, hematoma, dog ear deformity, or wound dehiscence, were found. The hypertrophic scar on the nonvermilion portion was reported in one patient. One patient underwent a second surgical procedure for further lip reduction. None of the patients or their parents complained of a worse appearance or the influence on functions. VAS evaluation showed a median value of 8.0 (range, 4.0–10.0), indicating a highly satisfactory outcome. In addition, there was no significant difference in VAS value between the upper and lower lip group (*P* = 0.327). The cases presented in Fig. 4 showed the efficacy of the SMISS approach in correcting deformity of upper or lower lip.

## Discussion

**Fig. 5:**
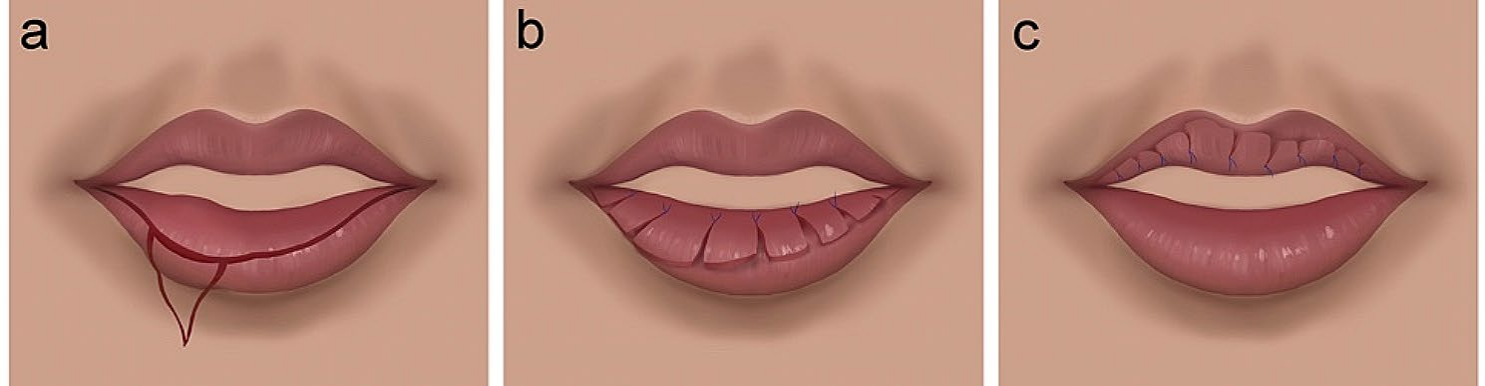
The schematic diagram of the pivotal steps in the lip reduction process for lip IH. **a**, The horizontal shortening of the lip. **b-c**, The vertical shortening of the lower or upper lip respectively

IHs occur with a high frequency in the central facial area [6] and nearly 20% of them have lip involvement [10]. Lip IHs are usually associated with functional, aesthetic, and psychosocial implications. In the recent decade, propranolol is considered a well-tolerated and effective drug that can keep IHs from rapid proliferation and can further shrink the existing lesions [11]. However, the obvious residue was still reported in some cases of lip IHs. Concerning the central position and symmetric pattern of the lips, even relatively minor remnants of the involuted IH are noticeable, which will lead to persistent deformity. A retrospective study reviewing 76 localized lip IHs demonstrated that only 10% reached complete regression and the remainder required subsequent treatment, the majority of which were advised to receive the surgical therapy [12]. The growth of IHs tends to cause soft tissue expansion, necessitating the volumetric reduction by surgical excisions. Besides, in contrast to oncologic resection which requires extended excision to achieve clear resection margins [13], normal size, natural contour, and improved appearance should be taken as the priority of the surgical process for lip IHs. Thus, to address the functional and aesthetic concerns of the patient and parents, we have modified the relevant surgical technique (Fig. 5).

The SMISS approach, first reported in 2017, is characterized by extensive undermining and multiple incisions, bringing favorable outcomes in patients with intermediate-to-large facial congenital melanocytic nevus [8, 14]. To the best of our knowledge, this study is the first to apply this approach in treating involuted lip IHs. In previous research regarding the surgical management of lip IH, the incisions were mostly confined to the lesions and the surrounding area [15, 16]. The local incision tends to offer a limited operative field, in which case the adjustment will be performed only in the unilateral unit of the upper or lower lip, making it difficult to achieve lip symmetry. However, a narrow tolerance for deformity of the lip is commonly seen in IH patients by their parents since the lip possesses highly specialized functions, including facial expression and oral competence. Generally, an aesthetic outcome of the lip reconstruction demands integral symmetry and a natural, tapering contour. We think that the modified surgical technique for lip IHs (developing from the SMISS approach) is more practical to achieve a satisfactory appearance. To be specific, the longer incision, covering the entire wet-dry junction, provides the opportunity to focus on integrity rather than the focal area. Meanwhile, the vermilion usually heals with imperceptible scarring, so the extended incision will not add the risk of surgery sequela. Then, the multi-incisions create several separate but adjustable flaps, which means the surgeon can stepwise regulate the length, orientation, and shape of each flap. In this manner, any distortion of the processed flap is clearly observed and could be corrected immediately. More importantly, the volume of each segment that needs to be resected can be dynamically adjusted so that over-reducing will be avoided, with appropriate vermilion volume remaining. Collectively, combined with the SMISS technique, we have offered a more promising approach to improving the aesthetic outcome for lip IH in a single-stage manner.

When confronted with the IH lesion increasing the horizontal dimension of the involved lip, we performed a full-thickness, pentagon-shaped resection with the vertical extended incision. Firstly, the full-thickness lip excision is used to prevent bunching of the subcutaneous and muscle layers. Secondly, the pentagonal block excision of the IH and its adjacent skin is preferred because it will only result in one single linear scar. The rectangular block excision, reported in the previous research, could lead to two intersecting scars [17]. Lastly, the extended incision can effectively prevent the dog-ear deformity, and it also allows the surgeon to preserve or rebuild the mentolabial sulcus.

In our center, the involuted IH on the cutaneous nonvermillion portion of the lip, namely erythema or telangiectasia, is not suggested to be completely resected. The major consideration is that the superficial lesions can be resolved by pulsed dye laser (PDL) [18], without visible scars remaining. It should be noted that PDL therapy can produce a permanent bleaching of the vermilion [17], so it’s not suitable for the vermilion area. Besides, we think that the lip symmetry takes priority while the resection of the nonvermillion portion could probably result in the distortion of philtrum or mentum.

In practice, what the parents are mostly concerned about is the optimal timing for the surgical treatment. In the early days, early excision was recommended for lip IHs [17]. However, in the propranolol era, a consensus has been reached that commencing oral propranolol in the proliferative phase is the first-line therapy [19]. Nowadays, surgery is preferable for select cases where medical management is contraindicated or unsuccessful. We agree that early treatment with propranolol can restrain the hemangioma growth and make it more amenable to surgery. Having surgery after infancy is more acceptable for the parents as well. In our center, propranolol withdrawal began when the patient was at least 12 months old and the tumor volume had not changed for 3 months, or when no tumor was detected by color Doppler ultrasound [20]. Therefore, we tend not to prolong the period of propranolol treatment for involuted IH when the drug effect has reached a plateau. Subsequently, further treatment (surgery, for instance) for the sequelae should be suggested. For another, both the aesthetic and functional impairment caused by lip IH will become a psychological burden in school-age patients. Thus, we usually advise them to receive surgery in the pre-school period.

Our study was limited in that the lower lip lesions (14/18) accounted for more than 70% of all eligible cases, which was probably caused by its retrospective design. However, several studies also found that lip IH occurred more commonly on the lower lip or on the mandibular segment [16], and lesions involving these sites had increased susceptibility to ulceration [21]. In our opinion, these findings can partly explain the high proportion of lower lip lesions in our study. The differences between the lower and the upper lip lesions might be owed to the different facial placode involvement of them [16]. Another limitation to this retrospective study was that patients’ photographs were the sole indicators used to measure primary outcome, in which the quality of photographs might have affected VAS scores. Thus, a prospective study involving a larger amount of lip IH cases is necessary, and multiple means of assessment should be applied.

## Conclusions

The present study demonstrates that applying the SMISS approach to the surgical management of involuted lip IH is practical and effective. In conclusion, this modified surgical technique can yield a cosmetically pleasant outcome, and it is, therefore, worth promotion in the future. The advancement in surgical techniques for lip IH is still necessary even in the propranolol era.

## Data Availability

The detailed clinical data of all patients that were analyzed during the current study are not publicly available due to the respect of participants’ rights to privacy and the protection of their identity but are available from the corresponding author on reasonable request.
